# Supplementary material for: Knockdown of Atg7 Induces Nuclear-LC3 Dependent Apoptosis and Augments Chemotherapy in Colorectal Cancer Cells
Source: Int J Mol Sci. 2020 Feb 7;21(3):1099. doi: 10.3390/ijms21031099 (PMC7038172; doi:10.3390/ijms21031099)
Supplement: Supplementary file 1 [file ijms-21-01099-s001.zip › ijms-660166-supp-revise-2/ijms-660166-supp-revise-2.pdf]

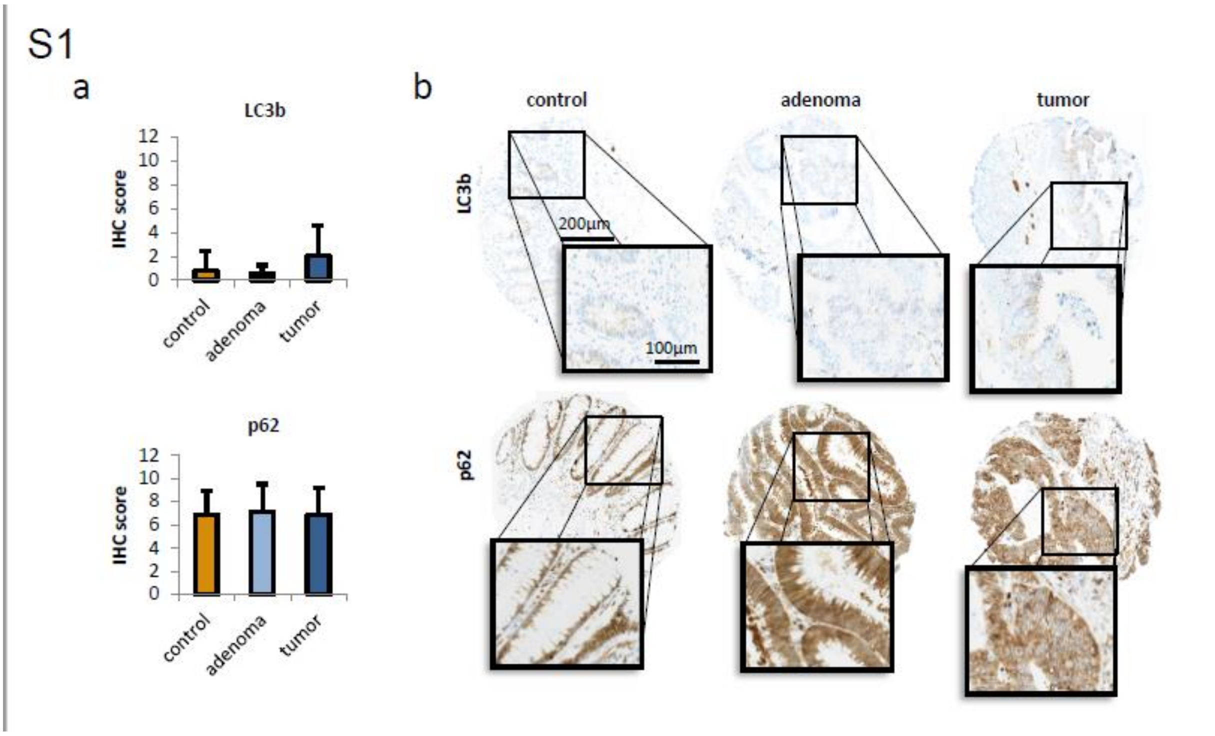

Figure S1. Expression levels of LC3b and p62 were unaltered in colorectal carcinogenesis. (a) Relative expression of autophagy-associated proteins LC3b and p62 in a tissue micro array (TMA) of non-matched human colon mucosa (n=10), adenoma (n=18) and carcinoma (n=49). Data represent mean + SD. **(b)** Representative images of LC3b (upper panel) and p62 (lower panel) staining on control (mucosa), adenoma and adenocarcinoma TMA cores. Scale bars as indicated.

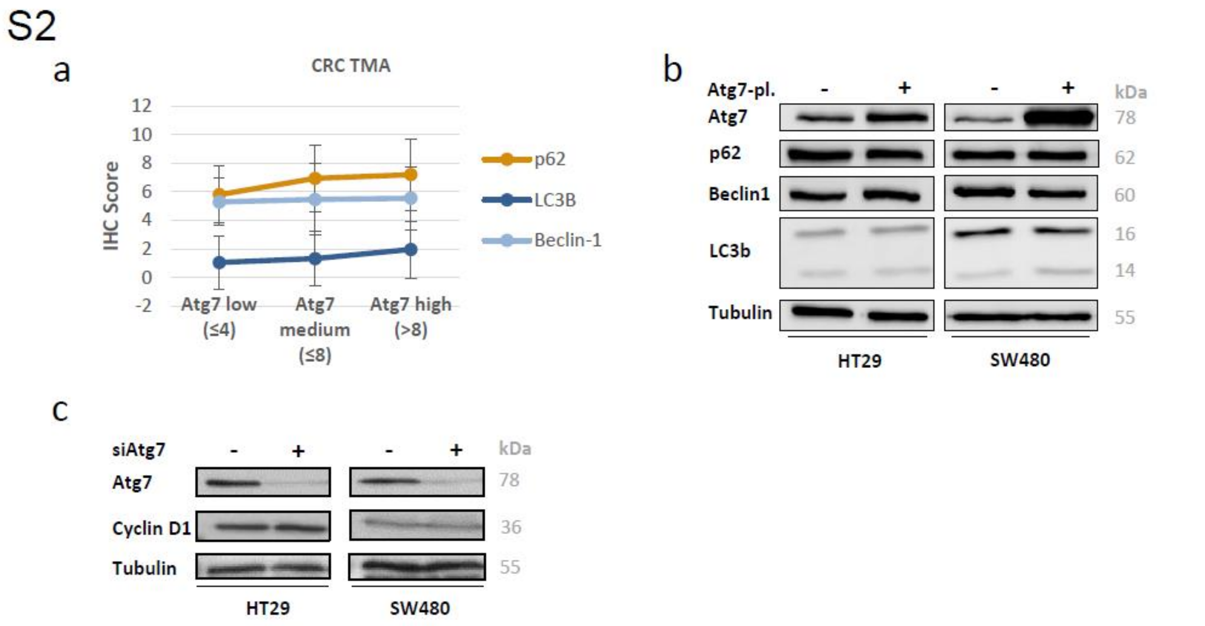

Figure S2. Expression of Beclin-1, p62 and LC3b was not significantly altered after overexpression of Atg7. (a) Mean IHC scores of Beclin-1, p62 and LC3b in Atg7 low (IHC score ≤4), Atg7 medium (IHC score ≤8) and Atg7 high (IHC score >8) expressing groups. Beclin-1: n=14 (Atg7 low), n=49 (Atg7 medium), n=31 (Atg7 high); p62: n=10 (Atg7 low), n=35 (Atg7 medium), n=25 (Atg7 high); LC3b: n=16 (Atg7 low), n=49 (Atg7 medium), n=31 (Atg7 high). Data represent mean ± SD. **(b)** Immunoblotting for key autophagic proteins in HT29 and SW480 cells, 48h post-transfection with a plasmid expressing

Atg7. **(c)** Immunoblotting for cell cycle protein Cyclin D1 in HT29 and SW480 cells, 48h after transfection with a siRNA targeting Atg7 (80nM).
