# Supplementary figures and images for: Knockdown of Atg7 Induces Nuclear-LC3 Dependent Apoptosis and Augments Chemotherapy in Colorectal Cancer Cells
Source: Int J Mol Sci. 2020 Feb 7;21(3):1099. doi: 10.3390/ijms21031099 (PMC7038172; doi:10.3390/ijms21031099)

a

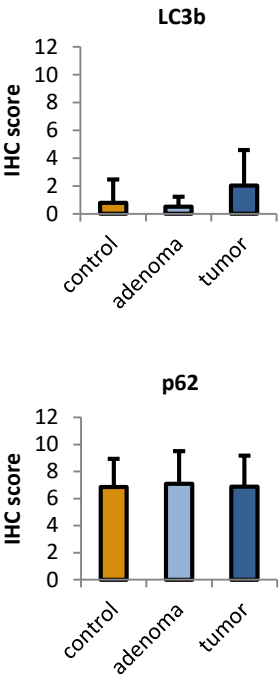

b

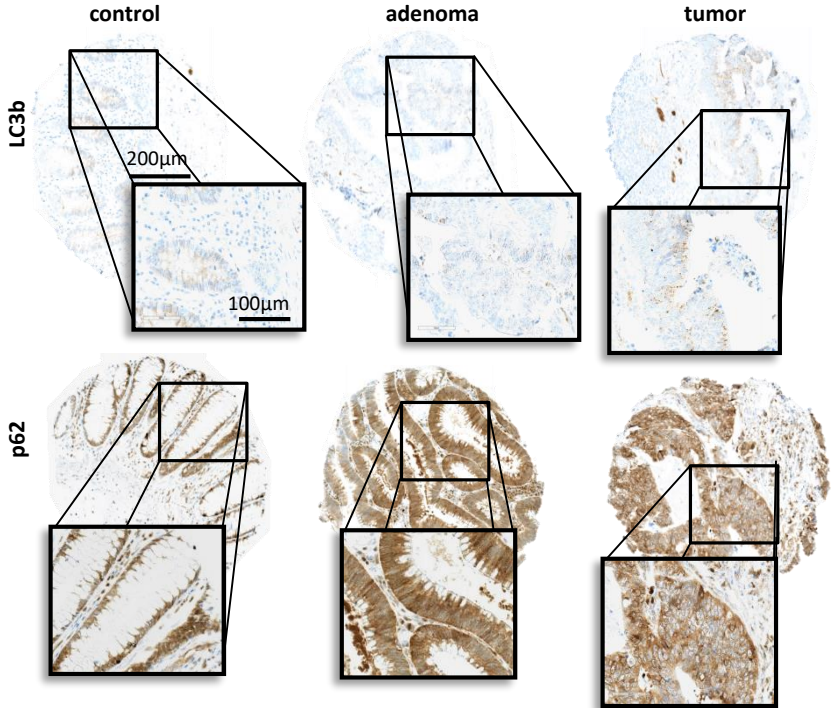

Supplement: Supplementary file 1 [file ijms-21-01099-s001.zip › ijms-660166-supp-revise-2/Figure_S1.pdf]

a

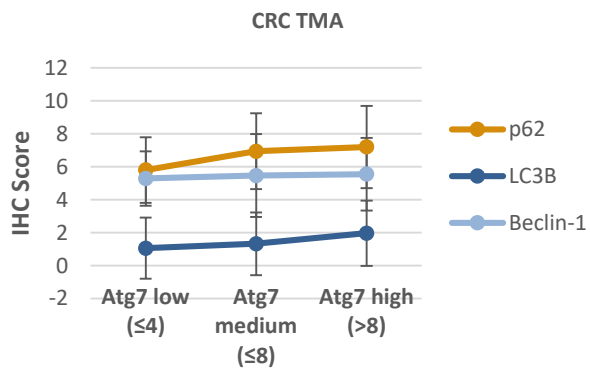

b

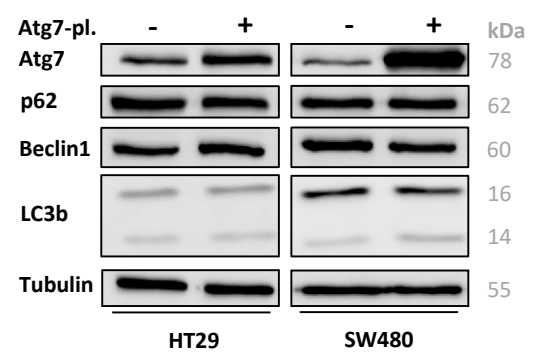

c

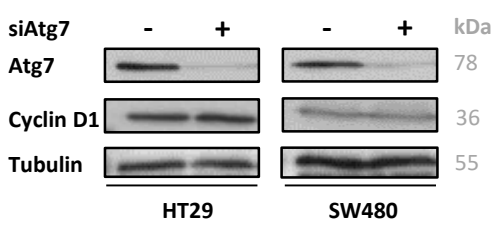

Supplement: Supplementary file 1 [file ijms-21-01099-s001.zip › ijms-660166-supp-revise-2/Figure_S2.pdf]
